# The use of tetanus post-exposure prophylaxis guidelines by general practitioners and emergency departments in the Netherlands: a cross-sectional questionnaire study

**DOI:** 10.1186/1471-2296-15-112

**Published:** 2014-06-09

**Authors:** Robine Donken, Nicoline van der Maas, Corien Swaan, Tjerk Wiersma, Margreet te Wierik, Susan Hahné, Hester de Melker

**Affiliations:** 1 Centre for Infectious Disease Control, National Institute for Public Health and the Environment, P.O. Box 1 (postbak 75), 3720 BA Bilthoven, The Netherlands; 2 Dutch College of General Practitioners, Utrecht, The Netherlands

**Keywords:** Tetanus, Post-exposure prophylaxis, Guidelines, Health council, General practitioners, Emergency departments

## Abstract

**Background:**

The Dutch National Immunisation Programme includes six tetanus toxoid (TT) vaccinations and reaches a high rate of vaccination coverage. In the Netherlands, several guidelines related to tetanus post-exposure prophylaxis (T-PEP) are in place. In 2003, the Dutch Health Council (HC) reviewed the use of T-PEP. The aim of this study is to evaluate whether the HC recommendations have been implemented.

**Methods:**

We asked 178 Dutch General Practitioner (GP) offices and 60 Emergency Departments (EDs) to participate in a cross-sectional questionnaire study and requested that participating facilities send in the T-PEP guidelines adopted by their practice. The differences, based on categories mentioned in the HC recommendations, between GPs and EDs and the type of T-PEP guidelines adopted were assessed.

**Results:**

The response rates for the GPs and EDs were 38% (n = 67) and 70% (n = 42), respectively. 98% percent (n = 107) of the participants reported having T-PEP guidelines. Of the guidelines described in the survey responses, 28% (n = 23; EDs 41%, GPs 21%) were consistent with the HC-recommendations, 36% (n = 29; EDs 7%, GPs 52%) adhered to the guidelines of the College of GPs (CGP), which restricts the use of T-PEP to tetanus prone wounds but for these wounds is in line with the recommendations of the HC. The remaining 36% had adopted other guidelines, most of which can lead to over-prescription of T-PEP. Information on T-PEP was lacking in patients with higher risk vaccination histories.

**Conclusion:**

Almost all participants have adopted T-PEP guidelines. Strict adherence to the HC recommendations is low. More than half of GPs have adopted the more restrictive CGP-guideline, which limits T-PEP to tetanus prone wounds.

## Background

Tetanus is a serious infectious disease that can be lethal when left untreated. Even treated, the disease’s mortality rate is between 10-40% [1]. Tetanus is caused by *Clostridium tetani* and is not contagious. Spores of the tetanus bacillus, which are present in the soil and in the faeces of domestic animals, can enter the body through a wound. Anaerobic conditions can then lead to production of the neurotoxin tetanospasmin, which causes muscle contractions and spasms [2,3].

Tetanus infection occurs worldwide, though there are only few cases each year in developed countries (including the Netherlands) due to large scale immunisation programmes [4,5]. The Netherlands started its National Immunisation Programme (NIP) in 1957. Currently, tetanus vaccinations are given at two, three, four and 11 months and at four and nine years of age [6]. Furthermore, in 2002, the NIP began administering Meningococcus serogroup C vaccines conjugated to tetanus toxoid (TT) to 14-month-old children and implemented a catch up campaign for those up to 19 years of age. This vaccination also boosts the immune response against tetanus [6,7]. In contrast to other countries, the Netherlands does not supply a regular booster for adults. As a result of continuously high NIP coverage, the seroprevalence of tetanus antibodies in the Netherlands is 94% [6].

High vaccination coverage is important because vaccination with TT is the only way to immunise against tetanus [7]. A full course of TT for never vaccinated wounded patients involves three vaccinations at months zero, one and seven. TT does not induce immunity immediately; after the second dose, it takes approximately two to four weeks to exceed the minimum level required for short-term protection (0.01 IU/ml). Therefore, human anti-tetanus immunoglobulin (TIG) is indicated for all persons at risk of contracting tetanus after being wounded if they are not (fully) vaccinated or are immuno-compromised [4].

The use of different protocols and the presumption that prophylaxis after injury might be unnecessary led to a 2003 review of the tetanus post-exposure prophylaxis (T-PEP) use by the Dutch Health Council (HC). The HC is an independent scientific advisory board, which advises the government. A multidisciplinary team of experts writes guidelines and advices based on latest evidence [8]. Therefore, the authors considered the HC recommendations as the reference standard. The only factors in determining whether to administer T-PEP, according to the HC guidelines, are a patient’s vaccination history and eligibility for the NIP, based on the year of birth (Table 1) [9]. The Dutch College of General Practitioners (CGP) states to use T-PEP only in tetanus prone wounds. For these wounds the CGP guideline is in line with the HC recommendations. The CGP guideline does not prescribe T-PEP in case of a non-tetanus prone wound. Differentiation between prone and non-prone wound types is determined by the likelihood that a wound is contaminated with *C. tetani* spores [10]. GPs differentiate the wound before consulting the patient’s vaccination history.

**Table 1:** The recommendations for tetanus post-exposure prophylaxis after injury published by the Dutch Health Council in 2003

**Vaccination history**	**Administration of TIG**	**Vaccination with TT**
Immuno-compromised	Yes	Three times
Never vaccinated	Yes	Three times
Incompletely vaccinated	Yes	Complete to three times
Fully vaccinated without documentation (men born before 1936 and women born before 1950)	Yes	One time
Fully vaccinated without documentation (men born in/after 1936 and women born in/after 1950)	No	One time
Fully vaccinated with booster ≥10 yrs ago.	No	One time
Fully vaccinated with booster <10 yrs ago.	No	No

TIG is administered (only) once. (TIG = anti-tetanus immunoglobulin, TT = tetanus toxoid).

Fully vaccinated patients completed the primary series of vaccinations (e.g., vaccinated with TT three times).

In 2011, five cases of tetanus were reported in the Netherlands. In three of those cases, patients contacted physicians for wound care, two directly after being injured and the other after two days because of an infection. In all three cases, T-PEP was not sufficient. It is therefore useful to evaluate whether the current recommendations related to T-PEP are properly implemented in clinical care [5]. The objective of this study is to assess whether the HC recommendations regarding T-PEP are in place and to evaluate the adoption of T-PEP guidelines that do not conform to the HC recommendations.

## Methods

### Participants

General practitioner (GP) offices and emergency departments (EDs) in the Netherlands were asked to participate in this cross-sectional study. At least five GPs were selected from each of the 12 provinces of the Netherlands. EDs were also chosen based on their distribution across the country. Potential participants were contacted by telephone, and a questionnaire was sent to the professionals who were willing to participate. A sample size calculation suggested that to estimate 20% (±10%) non-adherence (defined as aberrant for at least one aspect of the HC recommendations), with α = 0.05, at least 62 GPs and 40 EDs should be included in the study.

The study comprised on a short questionnaire resulting in a very limited burden to participants (i.e. professionals). For this reason no ethical approval was requested according to the Dutch Medical Research Involving Human Subjects Act (WMO) [11].

### Questionnaire

The questionnaire was sent either by post or by an email invitation to fill out an online questionnaire. Only one person per practice or department needed to fill out the questionnaire. The questions concerned the size of the practice, number of wounds observed and T-PEP treatments given, knowledge regarding the annual number of tetanus cases in the Netherlands, guidelines adopted at the responding facility and reasons for choosing those guidelines. Participants were asked to indicate whether they used no guidelines at all, the guidelines from the World Health Organization (WHO), the guidelines from the CGP, the guidelines from the LCI (based on the HC recommendations) or other guidelines. Additionally, the participants were asked to provide a copy of their guidelines or protocol related to T-PEP. The provided guidelines were scored according to their conformity with the HC recommendations, which were considered as the reference standard in this study.

### Analysis

Along with descriptive statistics, the T-PEP type was categorised based on the HC recommendations, and differences between the guidelines adopted by GPs and EDs were assessed. Finally, we determined the overall percentage of guidelines that conformed to the HC recommendations.

Statistical analyses were performed using SAS 9.3 for Windows (SAS Institute Inc., Cary, NC, USA).

## Results

In total, 178 GP offices and 60 EDs were contacted. Fifty-one GPs directly declined to participate in the study, whereas none of the approached EDs initially declined to participate. The reasons for non-participation varied, with GPs indicating that they were already too busy (45%, n = 23), not interested (26%, n = 13) or never participated in any research (16%, n = 8); seven GPs declined without providing a reason. Sixty GPs and 18 EDs did not return the questionnaire after initially agreeing to participate. In total, 67 GP offices and 42 EDs returned the questionnaire and participated in this study, resulting in response rates of 38% and 70%, respectively.

### General information on practices and tetanus (post-exposure prophylaxis)

#### General practitioners

The median number of GPs per practice was three (range 1–8), and the median number of patients per office was 3600 (range 1200–14700). All GPs stated that they occasionally prescribed and administered T-PEP in their offices (range <1 – 20 per month). Ninety-nine percent of the participating offices (n = 66) reported that T-PEP guidelines had been adopted. Only one of the GPs responded that no guidelines were in use, reporting that the guidelines for T-PEP were too complicated.

#### Emergency departments

The median number of patients visiting the responding EDs in 2011 was 22344 (range 1500–46000). The frequency of T-PEP administration at those EDs ranged from never to hundreds per week. 98% of the participating EDs (n = 41) reported that they had adopted T-PEP guidelines. Only one ED responded that no guidelines had been adopted because of limited access to the guidelines.

#### Knowledge regarding the number of tetanus cases

**Figure 1:**
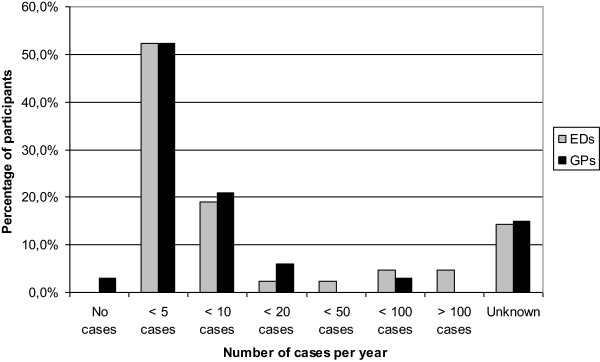
Participant estimations (by percentage) of the number of annual tetanus cases in the Netherlands.

The range of answers to the question of how many tetanus cases occurred annually in the Netherlands was quite large (between zero and more than one hundred). Sixteen participants stated that they had no idea of the number of cases. Eighty-five percent (n = 79) of the answers were between one and ten (Figure 1).

### Guidelines

**Figure 2:**
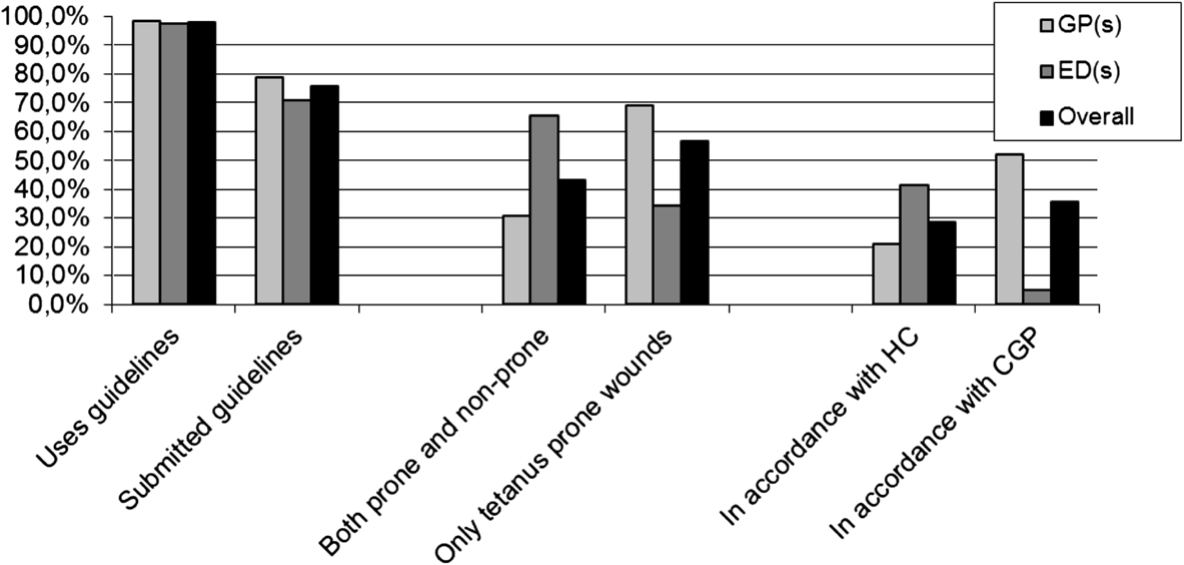
**Adoption of guidelines for tetanus post-exposure prophylaxis as reported by general practitioners (GPs) and emergency departments (EDs) in the Netherlands in 2012.** The guidelines of the CGP differs from the HC recommendations only in that it uses ‘type of wound’ as a criterion for determining whether T-PEP is appropriate. On all other criteria, the two guidelines are identical.

In total, 98% (n = 107; 66 GPs and 41 EDs) of the participating facilities had adopted T-PEP guidelines (Figure 2). Of the GP offices, 52 (78%) sent a copy of their guidelines. The guidelines provided conformed to HC recommendations and CGP guidelines (i.e., according to HC recommendations in Table 1 but only in the tetanus prone cases) in 11 (21%) and 27 (52%) cases, respectively. The remaining GPs (n = 14, 27%) used their own or other T-PEP guidelines; five of those GPs did not consider the wound type as a criterion in their guidelines, whereas nine did.

Of the EDs that had adopted T-PEP guidelines, 71% (n = 29) provided copies. Twelve EDs (41%) fully adhered to the HC recommendations. Two others (7%) conformed to the CGP guidelines (HC recommendations for tetanus prone wounds only). Two departments (7%) reported that they were not aware of the HC recommendations, although they used guidelines that were based partly on those recommendations (one ED used wound type as a criterion, and the other independently conformed). The remaining EDs (n = 15, 52%) had adopted their own T-PEP guidelines or other alternate guidelines; seven of those did not consider the wound type as a criterion, whereas eight did.

In facilities that used guidelines other than those based on the HC recommendations or the CGP guidelines (14 GPs and 15 EDs), non-adherence largely resulted in over-immunisation or missing information in certain categories of the vaccination history (Table 2).

**Table 2:** Absolute numbers (row percentages) of prescribed T-PEP in guidelines (n = 29) that were not based on the HC recommendations or CGP guidelines in 2012

**Vaccination history N (%)**	**No prophy-laxis**	**1x TT**	**1x TT + TIG**	**Complete TT till 3 + TIG**	**3x TT + TIG**	**No informa-tion**
***General practioners (n=14)***						
Immuno-compromised					* 10 (71)	4 (29)
Never vaccinated				1 (7)	* 12 (86)	1 (7)
Incompletely vaccinated				* 4 (29)	6 (42)	4 (29)
Fully vaccinated without documentation (men born before 1936 and women before 1950)			* 10 (71)			4 (29)
Fully vaccinated without documentation (men born in/after 1936 and women in/after 1950)		* 8 (57)			2 (14)	4 (29)
Fully vaccinated with booster ≥10 yrs ago.		* 12 (86)	1 (7)		1 (7)	
Fully vaccinated with booster <10 yrs ago.	* 12 (86)	2 (14)^1^				
***Emergency departments (n = 15)***						
Immuno-compromised					* 10 (67)	5(33)
Never vaccinated					* 15 (100)	
Incompletely vaccinated			2 (13)	* 1 (7)	7 (47)	5 (33)
Fully vaccinated without documentation (men born before 1936 and women before 1950)			* 10 (67)		2 (13)	3 (20)
Fully vaccinated without documentation (men born in/after 1936 and women in/after 1950)		* 9 (60)			3 (20)	3 (20)
Fully vaccinated with booster ≥10 yrs ago.		* 14 (93)	1 (7)^2^			
Fully vaccinated with booster <10 yrs ago.	* 14 (93)	1 (7)^1^				

When the guideline contained no information on T-PEP for a specific group, the guideline was scored as no information.

*Recommendations of the HC.

^1^When vaccinated longer than five years ago, a dose of TT is administered.

^2^When vaccinated longer than 15 years ago, both TT and TIG are administered.

## Discussion

Our survey studying the adherence to T-PEP guidelines showed that almost all participating GPs and EDs had adopted T-PEP guidelines. Less than one-third of the guidelines were fully consistent with the published HC recommendations, which are considered the reference standard (provided by a consensus of experts) [9]. Another 36% of the guidelines included the CGP suggestion to restrict the T-PEP use to tetanus prone wounds only, which might lead to under immunisation. The remainder of the adopted guidelines mainly resulted in over-immunisation when compared with the HC recommendations or provided inadequate information for specific categories of the vaccination history.

The percentage of Dutch GPs and EDs that had adopted T-PEP guidelines (98%) is somewhat higher than the 92% reported by Slottje et al. in 2001; that study took place before the HC review in 2003 and only included EDs [12].

The HC recommendations regarding T-PEP are based entirely on the patient’s vaccination history. TT and TIG are both recommended irrespective of the nature and origin of the wound [9]. By contrast, the CGP guidelines restrict the use of both TT and TIG to tetanus-prone wounds, such as those in contact with faeces, soil and dirt. The CGP notes that it is infeasible to obtain a patient’s vaccination status on every wound consultation due to high numbers of contacts [10]. In our study, 28% and 36% of responding Dutch health care facilities had adopted guidelines consistent with the HC recommendations and CGP guidelines, respectively. Since one might expect higher response of “best” performing (or highly interested practices) could lead to selection bias due to non-participation, these estimates might be an overestimation of the health care facilities which use a guideline for T-PEP in general and for the HC recommendation and CGP guidelines in specific.

Our study suggests that participating facilities that do not adhere to the HC recommendations or the CGP guidelines probably over-immunised (in)completely vaccinated patients, unnecessarily increasing the risk of side effects and costs [2,13]. However, in the categories for unvaccinated, probably vaccinated and immuno-compromised patients, a high percentage (20% to 33%) of guidelines not conforming to the HC recommendations contained no information on T-PEP prescriptions. Previous research on T-PEP among these specific groups has shown that increased tetanus risk coincided with an increased risk of under-prescription of T-PEP [13-15]. Under prescription might lead to a higher risk of developing tetanus. The consequences of a tetanus infection are still enormous. Even in developed countries, mortality is high and patients are often hospitalized for a long time.

The use of guidelines conforming to the CGP recommendations theoretically might result in a slightly higher risk of tetanus. However, the risk of tetanus contamination in a clean wound is very low, and the anaerobic conditions that are needed for spore germination are less likely to occur in superficial wounds [4,16-18]. On an individual basis, the risk of a patient visiting a facility using the CGP guideline to contract tetanus due to insufficient T-PEP is small.

However, vaccinating every wounded patient regardless of wound type also provides future protection from tetanus. This is consistent with the current British and American guidelines, which suggest evaluating the wound type and vaccination history simultaneously; anyone with incomplete, unknown or uncertain vaccination status and clean wounds is eligible for vaccination with TT to ensure future protection, as prophylaxis by TT takes time before boosting immunity [4,16,19]. For wounds that are considered tetanus prone, TIG is also administered [3,4].

The five Dutch tetanus case reports indicate that T-PEP was not adequately administered to elderly patients born before the NIP was introduced; three of these patients contacted physicians about wounds after falling on the street or stepping on a rusty nail, two did not seek wound care [5]. To ensure protection against tetanus for individuals born before the NIP was introduced, a catch-up campaign for the elderly could be considered. A quick calculation using Dutch data suggests that vaccinating every ten years all of the elderly born before 1951 (instead of maintaining the current strategy) would cost over five million euro per additional avoided tetanus case in the first ten years. In 2013, there were 3.454.500 people in the Netherlands born before 1951 [20]. If all receive a three dose series of TT (€5.43 per dose) administered at the GP (€4.34 per vaccination) the total campaign would cost €101.251.395,- [21,22]. The last years showed in this age group mean number of tetanus cases of two per year. Assuming an effectiveness of ten years this would prevent twenty cases. Therefore, a decennial tetanus booster for Dutch elderly is not likely to be cost-effective.

In this study, only presence of T-PEP guidelines was examined. Individual adherence of these guidelines was not examined. Previous research in other countries showed that compliance to T-PEP guidelines was poor [15,23,24]. One difficulty of adhering to the T-PEP guidelines is obtaining a patient’s appropriate vaccination history [13]. The patient reported history seems to be an unreliable method for acquiring a patient’s immune status [25-28]. The use of a Tetanus Quick Stick, a bedside test that determines a patient’s immune status in 10 minutes, seems to be a good alternative [15,26-33]. The price of such bedside test is €5.70 per test [33]. Further research investigating the applicability of this test in the Netherlands is currently underway; the study will also generate more insight regarding individual adherence to T-PEP guidelines in the Netherlands. [ NCT3530, Dutch Trial Registry, http://www.trialregister.nl/trialreg/admin/rctview.asp?TC=3530].

There are some limitations to this study. Facilities without T-PEP guidelines might have declined to participate, resulting in selection bias. Additionally, this study covers only a small sample of all facilities involved in emergency care in the Netherlands; the sampling procedures were not completely random. Therefore, we cannot be certain that the facilities included in the study were a representative sample. Finally, this study only examined whether health care facilities had adopted guidelines as well as which guidelines had been adopted. Compliance with the guidelines at these facilities was not studied; research in other countries suggests that actual adherence to T-PEP guidelines is poor [15,23,24].

## Conclusions

Almost all participating GPs and EDs had adopted guidelines for T-PEP. Forty-one percent of EDs and 28% of GP offices adopted guidelines fully consistent with the HC recommendations. Tetanus awareness is important for GPs and EDs, especially those treating incompletely vaccinated or unvaccinated patients.

## Abbreviations

CGP: College of General Practitioners; ED: Emergency Departments; GP: General Practitioner; HC: Health Council; NIP: National Immunisation Programme; TIG: Anti-tetanus immunoglobulin; T-PEP: Tetanus post-exposure prophylaxis; TT: Tetanus toxoid; WHO: World Health Organization; WMO: Medical Research Involving Human Subjects Act.

## Competing interests

The authors declare that they have no competing interests.

## Authors’ contributions

RD participated in the study design, data collection, data analysis and writing of the manuscript; HdM and NvdM were involved in the study design, data analysis and revision of the manuscript; CS, MtW and SH participated in the study design and revision of the manuscript; and TW contributed to the revision of the manuscript. All authors read and approved the final manuscript.

## Pre-publication history

The pre-publication history for this paper can be accessed here:

http://www.biomedcentral.com/1471-2296/15/112/prepub

## References

[B1] Federale overheidsdienst Volksgezondheid Vaccinatie tegen tetanus bij volwassenen https://www.zorg-en-gezondheid.be/uploadedFiles/NLsite_v2/Ziekten/Vaccinaties/Informatie_voor_vaccinatoren/vaccinatie_fiche_volw_tetanus_06032013(1).pdf

[B2] Borrow R Balmer P Roper MH WHO Module 3: Tetanus The immunological basis for immunization series 2006 Geneva, Switzerland: WHO Document Production Services

[B3] Mallick IH Winslet MC A review of the epidemiology, pathogenesis and management of tetanus Int J Surg 2004 2 2 109 112 10.1016/S1743-9191(06)60056-3 17462232

[B4] Rhee P Nunley MK Demetriades D Velmahos G Doucet JJ Tetanus and trauma: a review and recommendations J Trauma 2005 58 5 1082 1088 10.1097/01.TA.0000162148.03280.02 15920431

[B5] te Wierik MJM Hahné SJM van Ooik PC van Lier AMC Swaan CM Tetanusprofylaxe na verwonding. Check de indicatie voor vaccinatie én immunoglobuline Ned Tijdschr Geneeskd 2013 157 A5906 24330787

[B6] Steens A Mollema L Berbers GA van Gageldonk PG van der Klis FR de Melker HE High tetanus antitoxin antibody concentrations in the Netherlands: a seroepidemiological study Vaccine 2010 28 49 7803 7809 10.1016/j.vaccine.2010.09.036 20875496

[B7] de HE M van den Hof S Berbers GA Nagelkerke NJ Rumke HC Conyn-van Spaendonck MA A population-based study on tetanus antitoxin levels in The Netherlands Vaccine 1999 18 1–2 100 108 10501240 10.1016/s0264-410x(99)00186-3

[B8] Gezondheidsraad http://www.gr.nl

[B9] Gezondheidsraad Immunisatie tegen tetanus bij verwonding 2003 Den Haag: Gezondheidsraad

[B10] Boukes FS Wiersma TJ Beaujean D Burgmeijer RJ Timen A Tetanusprofylaxe in de huisartsenpraktijk Ned Tijdschr Geneeskd 2004 148 44 2172 2173 15559410

[B11] Verenigde Commissies Mensgebonden Onderzoek Niet WMO plichtig onderzoek https://www.vcmo.nl/wmo/niet-wmo-plichtig-onderzoek/

[B12] Slottje P van Leeuwen Y Toepassing van passieve immunisatie bij tetanus 2001 Thesis: Katholieke Universiteit Nijmegen

[B13] Alagappan K Pulido G Caldwell J Abrahamian FM Physician compliance with tetanus guidelines for admitted versus discharged patients South Med J 2006 99 3 234 238 10.1097/01.smj.0000202705.51101.e3 16553097

[B14] Barjat C Charles R Lucht F Frappe P Gestion du risque tetanique des plaies en medecine generale Med Mal Infect 2011 41 8 424 429 10.1016/j.medmal.2010.12.014 21429681

[B15] Talan DA Abrahamian FM Moran GJ Mower WR Alagappan K Tiffany BR Pollack CV Steele MT Dunbar LM Bajani MD Weyant RS Ostroff SM Tetanus immunity and physician compliance with tetanus prophylaxis practices among emergency department patients presenting with wounds Ann Emerg Med 2004 43 3 305 314 10.1016/j.annemergmed.2003.09.017 14985655

[B16] Department of Health Chapter 30: Tetanus The Green Book 2009 United Kingdom: The Stationary Office

[B17] Center for Disease Control and Prevention Tetanus surveillance – United States, 2001–2008 MMWR Morb Mortal Wkly Rep 2011 60 12 365 369 21451446

[B18] Farrar JJ Yen LM Cook T Fairweather N Binh N Parry J Parry CM Tetanus J Neurol Neurosurg Psychiatry 2000 69 3 292 301 10.1136/jnnp.69.3.292 10945801 PMC1737078

[B19] Parker M Emergency nurse practitioner management of tetanus status and tetanus-prone wounds Int Emerg Nurs 2008 16 4 266 271 10.1016/j.ienj.2008.05.009 18929345

[B20] Centraal Bureau voor de Statistiek http://www.cbs.nl

[B21] Medicijnkosten http://www.medicijnkosten.nl

[B22] Kosten huisartsenzorg 2013 http://www.nza.nl/98174/139255/858862/TB-CU-7076-02_Tariefbeschikking_Huisartsenzorg.pdf

[B23] Savage EJ Nash S McGuinness A Crowcroft NS Audit of tetanus prevention knowledge and practices in accident and emergency departments in England Emerg Med J 2007 24 6 417 421 10.1136/emj.2007.047399 17513539 PMC2658278

[B24] Yoon YH Moon SW Choi SH Cho YD Kim JY Kwak YH Clinician awareness of tetanus-diphtheria vaccination in trauma patients: a questionnaire study Scand J Trauma Resusc Emerg Med 2012 20 35 10.1186/1757-7241-20-35 22587533 PMC3487935

[B25] Cooke MW Are current UK tetanus prophylaxis procedures for wound management optimal? Emerg Med J 2009 26 12 845 848 10.1136/emj.2008.070268 19934122

[B26] Paulke-Korinek M Rendi-Wagner P Kundi M Tomann B Wiedermann U Kollaritsch H Pretravel consultation: rapid dipstick test as a decision guidance for the application of tetanus booster vaccinations J Travel Med 2008 15 6 437 441 10.1111/j.1708-8305.2008.00252.x 19090799

[B27] Stubbe M Swinnen R Crusiaux A Mascart F Lheureux PE Seroprotection against tetanus in patients attending an emergency department in Belgium and evaluation of a bedside immunotest Eur J Emerg Med 2007 14 1 14 24 10.1097/01.mej.0000228449.37974.7e 17198321

[B28] Gindi M Oravitz P Sexton R Shpak M Eisenhart A Unreliability of reported tetanus vaccination histories Am J Emerg Med 2005 23 2 120 122 10.1016/j.ajem.2004.03.015 15765327

[B29] Colombet I Saguez C Sanson-Le Pors MJ Coudert B Chatellier G Espinoza P Diagnosis of tetanus immunization status: multicenter assessment of a rapid biological test Clin Diagn Lab Immunol 2005 12 9 1057 1062 16148171 10.1128/CDLI.12.9.1057-1062.2005 PMC1235798

[B30] Elkharrat D Sanson-Le-Pors MJ Arrouy L Beauchet A Benhamou F Evaluation of a bedside immunotest to predict individual anti-tetanus seroprotection: a prospective concordance study of 1018 adults in an emergency department Emerg Med J 2010 27 1 36 42 10.1136/emj.2008.068254 20029005

[B31] Hatamabadi HR Abdalvand A Safari S Kariman H Dolatabadi AA Shahrami A Alimohammadi H Hosseini M Tetanus Quick Stick as an applicable and cost-effective test in assessment of immunity status Am J Emerg Med 2011 29 7 717 720 10.1016/j.ajem.2010.01.046 20825874

[B32] McVicar J Should we test for tetanus immunity in all emergency department patients with wounds? Emerg Med J 2012 30 3 177 179 22685251 10.1136/emermed-2012-201193

[B33] Stubbe M Mortelmans LJ Desruelles D Swinnen R Vranckx M Brasseur E Lheureux PE Improving tetanus prophylaxis in the emergency department: a prospective, double-blind cost-effectiveness study Emerg Med J 2007 24 9 648 653 10.1136/emj.2007.048520 17711944 PMC2464632

